# Ultrasound-Guided Photoacoustic Monitoring of Vascular Ischemia in Implant-Induced Skin Necrosis

**DOI:** 10.1016/j.ultrasmedbio.2025.08.009

**Published:** 2025-09-12

**Authors:** Xinyue Huang, Anthony M. Yu, Samuel M.A. Morais, Jeong Hun Park, David A. Zopf, Scott J. Hollister, Stanislav Y. Emelianov

**Affiliations:** a Wallace H. Coulter Department of Biomedical Engineering, Georgia Institute of Technology and Emory University School of Medicine, Atlanta, GA, USA; b School of Electrical and Computer Engineering, Georgia Institute of Technology, Atlanta, GA, USA; c Department of Otolaryngology - Head and Neck Surgery, University of Wisconsin School of Medicine and Public Health, Madison, WI, USA

**Keywords:** High-frequency ultrasound, Photoacoustic imaging, Subcutaneous implant, Skin, Vascular Imaging

## Abstract

**Objective:**

Implant-induced skin necrosis remains a significant clinical challenge in subcutaneous implant applications, believed to result from vascular ischemia caused by mechanical stress. However, this hypothesis remains largely qualitative and lacks direct quantitative validation. This study investigates the potential of ultrasound-guided photoacoustic (US/PA) imaging as a non-invasive diagnostic tool for detecting vascular changes and predicting sites of breakdown in response to subcutaneous implants.

**Methods:**

Three designs of 3-D-printed porous poly-ɛ-caprolactone implants – a unimodal cube, a bimodal cube and a unimodal dome – were implanted subcutaneously in 16 SKHl-Elite hairless mice. Longitudinal US/PA imaging was performed bi-weekly for 12 weeks to monitor PA vasculature (532 nm), vascular density (532 nm) and total hemoglobin levels (700–950 nm). Skin health was scored using a modified National Pressure Injury Advisory Panel-based system. Temporal and spatial analyses were conducted to evaluate the relationship between PA-derived biomarkers and skin health deterioration.

**Results:**

Mice with implant exposure (N = 5) exhibited a progressive decrease in PA biomarkers, with reductions of up to 48% by week 12. These changes preceded visible skin necrosis by approximately 2–4 weeks. In contrast, the healthy group (N = 11) maintained stable signals. PA vasculature at 532 nm showed the most significant temporal differentiation (*p* = 0.0013) and strong correlation with skin health (R >0.85). Spatial hemoglobin maps revealed that local reductions in vascularity preceded localized skin dehiscence.

**Conclusion:**

Ultrasound-guided PA imaging enables early, non-invasive detection of vascular compromise associated with implant-induced skin necrosis. Among the tested biomarkers, PA vasculature at 532 nm emerged as the most sensitive and practical for early detection, offering a promising tool for guiding implant design and improving post-implantation monitoring.

## Introduction

Subcutaneous implants, especially those made from porous polyethylene, have been used extensively in clinical applications such as facial reconstruction and auricular repair [1–5]. These implants offer several advantages over traditional autologous grafts, including reduced surgical complexity, improved reproducibility of 3-D anatomical structures and better suitability for earlier intervention in pediatric cases, where there is limited donor costal cartilage [6–9]. Additionally, implants eliminate the need for secondary tissue harvesting, thereby reducing associated morbidities and patient burden [10]. Poly-ɛ-caprolactone (PCL), a thermoplastic polymer with excellent printability, is widely used in 3-D printing of medical devices and tissue regeneration scaffolds, and has demonstrated potential for clinical translation [11,12]. Despite these benefits, implant-induced skin necrosis and exposure remain significant challenges, often leading to infection and implant failure [13–15]. Implant exposure is a common complication across various facial implant procedures, occurring in nasal [16], craniofacial skeletal [17], and auricular implants. In these cases, implant exposure frequently necessitates revision surgeries with complex skin flaps, burdening patients and families, increasing healthcare costs, and negatively impacting patient outcomes. Similarly, in spinal surgery, skin dehiscence over scoliosis fixation hardware occurs in 2–20% of cases, often resulting in implant exposure, multiple reoperations, and long-term disability [18,19]. Dehiscence risks limit surgical options for all these clinical uses, for example making rib grafts the preferred surgical option for auricular reconstruction, despite the advantages offered by porous polyethylene implants [7,20,21].

An improved, quantitative understanding of the mechanisms underlying implant-induced skin necrosis is essential for early detection and the development of effective mitigation strategies. While previous studies have suggested that excessive mechanical stress on the skin contributes to vascular compromise and subsequent skin necrosis, these hypotheses remain largely qualitative and lack direct quantitative validation [22–26]. Cugno and Bulstrode [22] noted that the tight adherence of the thin skin envelope to rigid implant contours creates high mechanical stress, increasing the risk of vascular compromise and implant exposure if not addressed early. Zhong et al. [23] stated that the mechanical interaction between the skin and implant structure remains insufficiently understood, highlighting the need for optimization to reduce complications in auricular reconstruction. Although qualitative hypotheses suggest high skin stress leads to vascular ischemia, no quantitative relationship between implant-induced skin stress, vascular ischemia and damage has been reported. Establishing this connection is crucial for enhancing implant design, improving post-implantation monitoring and preventing complications associated with subcutaneous implants.

Multiple medical imaging techniques have been applied for vasculature imaging of the skin [27,28]. Laser speckle contrast imaging and laser Doppler perfusion imaging are optical imaging techniques used to assess blood flow with high resolution in superficial tissues [29–31]. However, both have limited penetration depth (~1 mm) and provide only relative blood flow or perfusion, making them unsuitable for quantitative measurements of vascular ischemia or future clinical translation [32]. Similarly, Doppler ultrasound imaging can detect blood flow at significantly greater depths but has low sensitivity for slow capillary blood flow and still lacks absolute quantification [33,34]. Contrast-enhanced ultrasound uses microbubble contrast agents to quantify blood volume and perfusion but requires contrast agent injection [35,36], which may alter physiological conditions and affect the mechanical properties of the skin. Photoplethysmography is a simple and widely used method for assessing vascular signals but has low spatial resolution and is limited to superficial, bulk measurements, making it inadequate for detecting localized ischemic changes [37]. Optical coherence tomography angiography has also been utilized for high-resolution, label-free visualization of skin microvasculature [38,39]. While optical coherence tomography angiography provides detailed structural information on microvascular networks, it lacks direct quantification of blood volume and is challenging to apply to human skin due to its limited imaging depth (1–2 mm).

Photoacoustic (PA) imaging is a hybrid imaging modality that combines the high spatial resolution of ultrasound with functional information derived from optical absorption of tissues. PA imaging works by utilizing pulsed laser excitation to induce thermoelastic expansion in biological tissues, generating ultrasound waves that are detected and spatially reconstructed [40–42]. As hemoglobin is a strong optical absorber, PA imaging can provide direct, label-free measurements of blood volume, oxygenation, microcirculation and vascular structure at depths greater than conventional optical imaging methods [41,43–45]. Ultrasound-guided PA (US/PA) imaging has been effectively utilized in various skin vasculature applications, including the non-invasive visualization of cutaneous microvasculature and the assessment of skin lesions [46,47]. Accordingly, US/PA imaging is particularly advantageous for evaluating implant-induced skin vascular compromise as it allows for non-invasive, real-time monitoring of changes to the skin vasculature overlying subcutaneous implants. It enables the assessment of early ischemic events, which are critical for predicting and preventing skin necrosis. Furthermore, US and PA images are inherently co-registered, ensuring accurate anatomical and functional correlation of vascular changes with tissue structure [48,49]. These capabilities make it a good prospective imaging tool for evaluating implant-induced vascular ischemia and its role in skin health deterioration.

This study aimed to investigate the relationship between vascular ischemia and implant-related skin necrosis in a mouse model with subcutaneous implants. Using 3-D US/PA imaging, we measured spatial-temporal changes in skin vascularity overlying the implant in response to different implant designs. We hypothesized that high skin stress, induced by implant geometry, leads to vascular ischemia, which can be detected *via* US/PA imaging, preceding visible skin atrophy, necrosis and implant exposure. By quantifying the changes in PA-derived biomarkers, this study sought to provide critical insights into the mechanisms of implant-induced skin necrosis and to inform strategies for early detection and prevention of implant exposure.

## Materials and methods

### Study design

**Figure 1.**
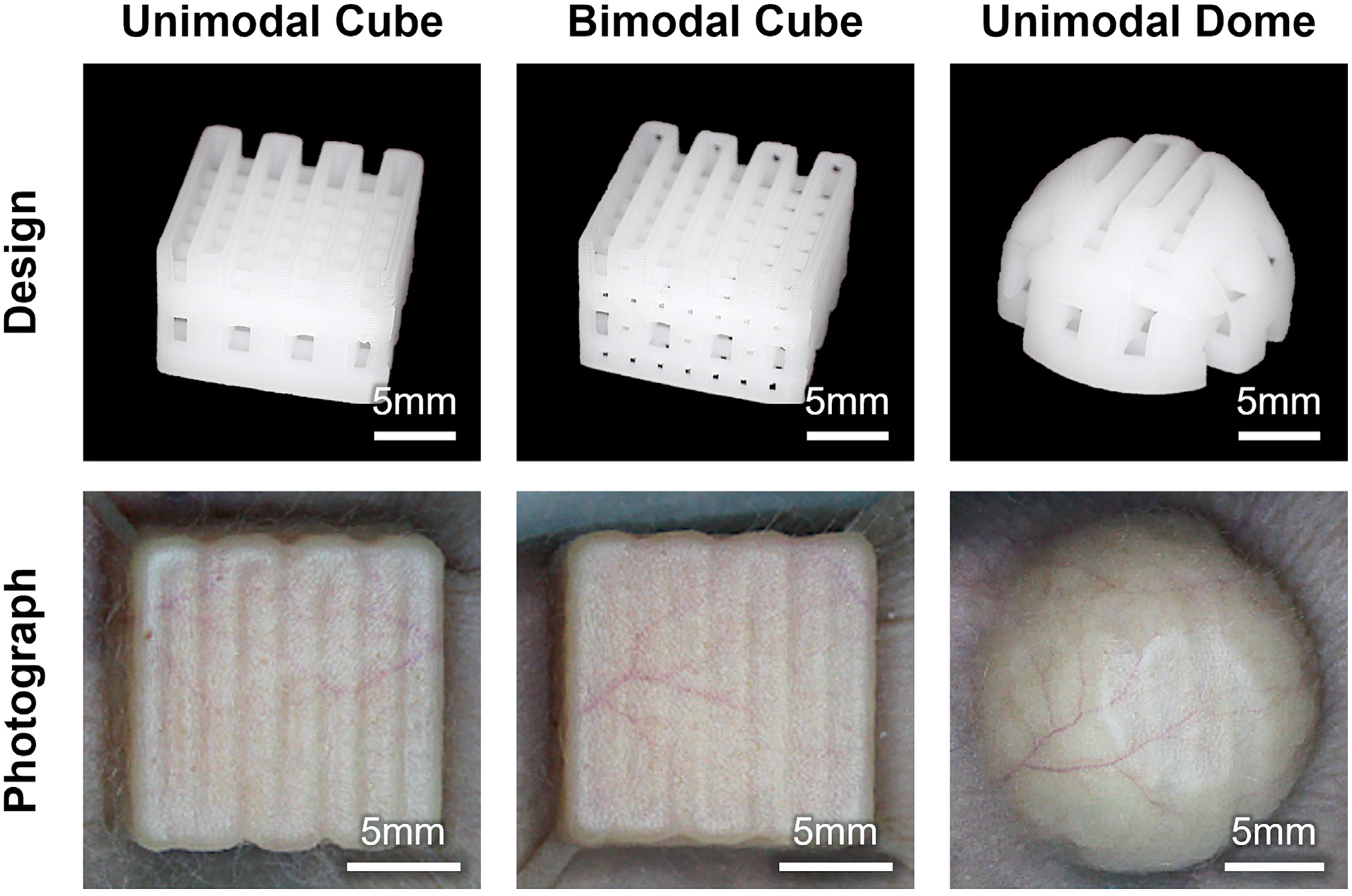
Illustration of the unimodal cube, bimodal cube and unimodal dome poly-ɛ-caprolactone porous implants, along with their corresponding post-implantation photographs.

Three PCL implants were designed and 3-D printed: a unimodal cube (single pore size) with dimensions of 14 × 14 × 9.6 mm (length, width, height), a bimodal cube (two pore sizes) with the same dimensions, and a unimodal dome with a diameter of 18 mm and a height of 9.6 mm. All the implants were designed to have a similar volume. The implants were fabricated using extrusion-based 3-D printing with medical-grade PCL (Evonik, C209). This grade of PCL complies with ISO 10993 standards required by the US Food and Drug Administration, ensuring biocompatibility and facilitating future regulatory approval. The cube and dome shapes were selected to represent two extremes of stress distribution: the cube, with its sharp corners and edges, was intended to simulate high-stress conditions similar to those observed in fractured implants, while the dome was designed to maintain broad skin contact and minimize stress concentrations. Implant sizes were chosen to be anatomically appropriate for mouse skin, with the dome implant designed to minimize adverse skin health outcomes and the cube implants designed to induce localized skin stress sufficient to trigger vascular ischemia. The bimodal cube implant, with increased porosity, was designed to promote enhanced tissue and vascular ingrowth, and we hypothesized that this design would improve skin health outcomes. The implant designs are illustrated in Figure 1. A total of 16 SKHl-Elite hairless mice (N = 8 female, N = 8 male) were used in this study and divided into four experimental groups based on implant type: unimodal cube, bimodal cube, unimodal dome and a control group without implants. Each group consisted of two male and two female mice.

### Implantation and imaging procedure

All animal procedures were approved by the Institutional Animal Care and Use Committee at the Georgia Institute of Technology in accordance with federal guidelines for the care and use of laboratory animals. Mice were anesthetized using isoflurane prior to implantation. A dorsal incision was made at the back of the neck to create a subcutaneous pocket and one of the three implants was inserted into the pocket with its top ridges oriented along the transverse direction and advanced caudally until it was fully positioned. The incision was offset to avoid direct overlapping with the implant. Ketoprofen was administered for analgesia, and the incision was sutured and sterilized. After surgery, a syringe was used to remove excess air between the skin and implant to ensure close tissue contact. Figure 1 presents post-implantation photographs of the three implant designs.

Three-dimensional US/PA imaging was performed to assess the skin overlying the implant. Imaging was conducted using a US/PA imaging system (Vevo LAZR, Fujifilm VisualSonics, Inc., Toronto, ON, Canada) equipped with a US/PA imaging probe (LZ-250, Fujifilm VisualSonics, Inc.) operating at a center frequency of 21 MHz. The Nd:YAG laser system (20 Hz, 5–7 ns pulse length) was used at 532 nm with a laser fluence of 5.4 mJ/cm^2^, and an Nd:YAG-pumped optical parametric oscillator tunable laser system was used within the 680–950 nm wavelength range with a fluence range of 4.1–6.8 mJ/cm^2^. The laser fluence was calibrated and optimized before each imaging session. A thin layer of ultrasound coupling gel was applied to the skin surface and a water bag was placed above the skin. The ultrasound transducer was submerged in the water to allow free movement without displacing the skin, ensuring high-resolution 3-D imaging. The imaging volume covered a 23 × 23 × 20 mm (X, Y, Z) region with a voxel size of 0.05 × 0.23 × 0.05 mm (X, Y, Z). 3-D US/PA imaging was performed at seven wavelengths (532, 700, 750, 800, 850, 900 and 950 nm) and raw US and PA image data were stored for further analysis.

The first imaging session was performed immediately post-implantation (week 0). Subsequent imaging sessions were conducted at weeks 2, 4, 6, 8, 10 and 12, following the same protocol to ensure longitudinal monitoring of skin vascular changes. To complement US/PA imaging, photographs of the skin over the implant site were captured using a Nikon digital camera prior to each imaging session. Skin health was visually assessed from these photographs using a five-point scoring system adapted from the pressure injury staging system established by the National Pressure Injury Advisory Panel [50,51]: 5 – no visible skin damage; 4 – minor spots or blisters with intact skin; 3 – small skin dehiscence without implant exposure; 2 – moderate dehiscence with slight implant exposure; 1 – severe dehiscence with significant implant exposure.

### Data processing

The collected 3-D US and PA raw data were first imported into Vevo LAB. The Vevo LAB spectral unmixing tool was used to process 3-D PA data acquired at 700, 750, 800, 850, 900 and 950 nm to generate 3-D maps of deoxygenated hemoglobin (Hb) and oxygenated hemoglobin (HbO_2_). The US data, PA data and unmixed Hb and HbO_2_ data were then exported to MATLAB for further analysis. All 3-D datasets were interpolated to achieve a uniform voxel size of 0.1 × 0.1 × 0.1 mm (X, Y, Z) for subsequent processing.

Based on the 3-D US data, a thresholding algorithm was applied to differentiate the hyperechoic regions of the mouse skin and underlying tissue from the anechoic regions of the surrounding water, allowing identification of the water–skin interface. Morphological image processing was then used to erode a 0.5 mm layer from the water–skin interface, corresponding to the average skin thickness along the Z axis. The skin region was segmented by subtracting the eroded volume from the original volume, generating the final skin segmentation. To isolate the horizontal (X-Y plane) extent of the skin overlying each implant, a 14 × 14 mm square region was manually selected over the unimodal and bimodal cube implants and an 18 mm diameter circular region was selected over the unimodal dome implants. For control animals, the entire 23 × 23 mm region was used. As US and PA data are inherently co-registered, the same segmentation was directly applied to the PA data, ensuring accurate localization of the overlying skin. To account for day-to-day fluctuations in laser fluence, PA amplitudes within the segmented skin region were normalized to the PA amplitude of the surrounding water.

Maximum intensity projection was performed on segmented PA volumes to convert the 3-D data into 2-D maps of the skin region. To suppress noise and enhance major vascular structure, the PA maps at 532 nm were normalized to a 0–1 scale, and the Jerman 2-D Hessian-based vesselness filter was applied [52,53]. The vessel scale parameter was set to 4, based on the measured size of the blood vessels. The resulting map represents an enhanced vascular structure map, scaled from 0 to 1. In some subjects, multiple vertical ridges were observed in the PA maps, which may have been misclassified as vessels by the filter. These ridges likely resulted from PA signals originating from the implant surface that were not fully segmented out (this finding is further discussed in the Discussion section). As most major blood vessels are anatomically oriented in the horizontal direction, we developed an algorithm to suppress these vertical artifacts. Specifically, we applied a Hessian-based Frangi vesselness filter [54] to determine the direction of the enhanced vessels. Any region in the Jerman-enhanced vascular map with a vessel direction close to vertical (angle between −0.5 and 0.5 radians) was set to zero.

### Data analysis

Three PA-derived biomarkers of skin vascularity were estimated as follows. First, PA vasculature, defined as the average amplitude of the PA signal at 532 nm within the skin region, was selected due to the strong optical absorption of hemoglobin and negligible absorption by water at this wavelength, which allows the PA signal at 532 nm wavelength to be highly sensitive to blood content in superficial tissues. While PA amplitude alone is not inherently quantitative, its relative changes over time can serve as an indicator of vascular integrity. Second, vascular density, calculated as the mean value of the Jerman-enhanced vascular structure map derived from the 532 nm PA data, was used to estimate the spatial density of major blood vessels in the skin region, thereby enhancing sensitivity to changes in major vascular architecture. Third, total hemoglobin level, defined as the mean of the sum of Hb and HbO_2_ levels, captured both arterial and venous contributions to the vascular supply.

For each subject, the PA vasculature, vascular density and total hemoglobin levels were calculated over time. These values were normalized to the baseline (week 0) to evaluate vascular changes in the skin post-implantation and to assess the progression of vascular ischemia. Based on the skin health outcomes, subjects were categorized into two groups: a healthy group, in which the skin overlying the implant remained intact at week 12, and an exposed group, in which implant exposure occurred by week 12. For each PA biomarker, normalized values were statistically analyzed using a linear mixed effects model. The model included fixed effects for time, PA biomarker levels and their interaction, with subject-specific random interceptions to account for repeated measurements. An analysis of variance was performed on the fitted model to evaluate the significance of differences between the healthy and exposed groups.

To investigate the temporal relationship between PA-derived biomarkers and skin health, time series of PA vasculature, vascular density and total hemoglobin levels were correlated with skin health scores. Cross-correlation analysis with lag estimation was performed to determine the maximum correlation coefficients and their corresponding time lags, where a negative lag with a high correlation coefficient suggested that PA signals could predict skin dehiscence. Single-sample *t*-tests were conducted to determine whether these correlation coefficients were significantly greater than zero, supporting a consistent positive relationship between PA biomarkers and skin health across subjects.

To analyze the spatial correlation between vascular ischemia and skin necrosis, segmented 3-D maps of total hemoglobin levels from the unimodal and bimodal cube groups were flattened into 2-D hemoglobin maps. Pixel-wise changes in hemoglobin levels were computed by normalizing each map to its mean value, applying Gaussian smoothing and subtracting consecutive time points to visualize hemoglobin reduction maps. These maps were then compared with the corresponding skin photographs to evaluate the spatial correspondence between hemoglobin loss and local skin deterioration.

## Results

### Skin health and PA biomarkers

**Figure 2.**
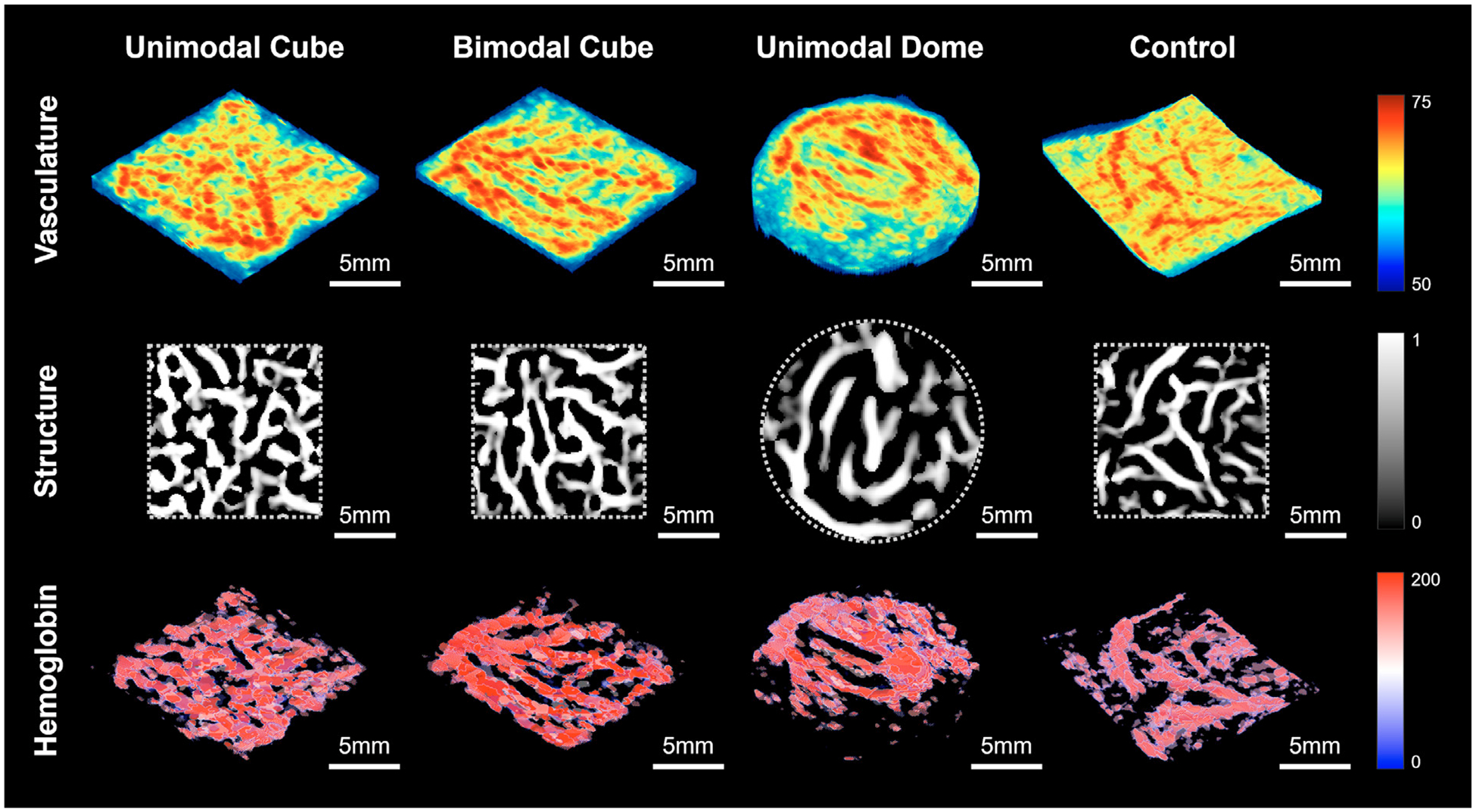
Segmented (*top row*) 3-D maps of photoacoustic vasculature, (*middle row*) 2-D map of the enhanced vascular structure, and (*bottom tow*) 3-D maps of total hemoglobin of the unimodal cube, bimodal cube, unimodal dome and control groups.

**Figure 3.**
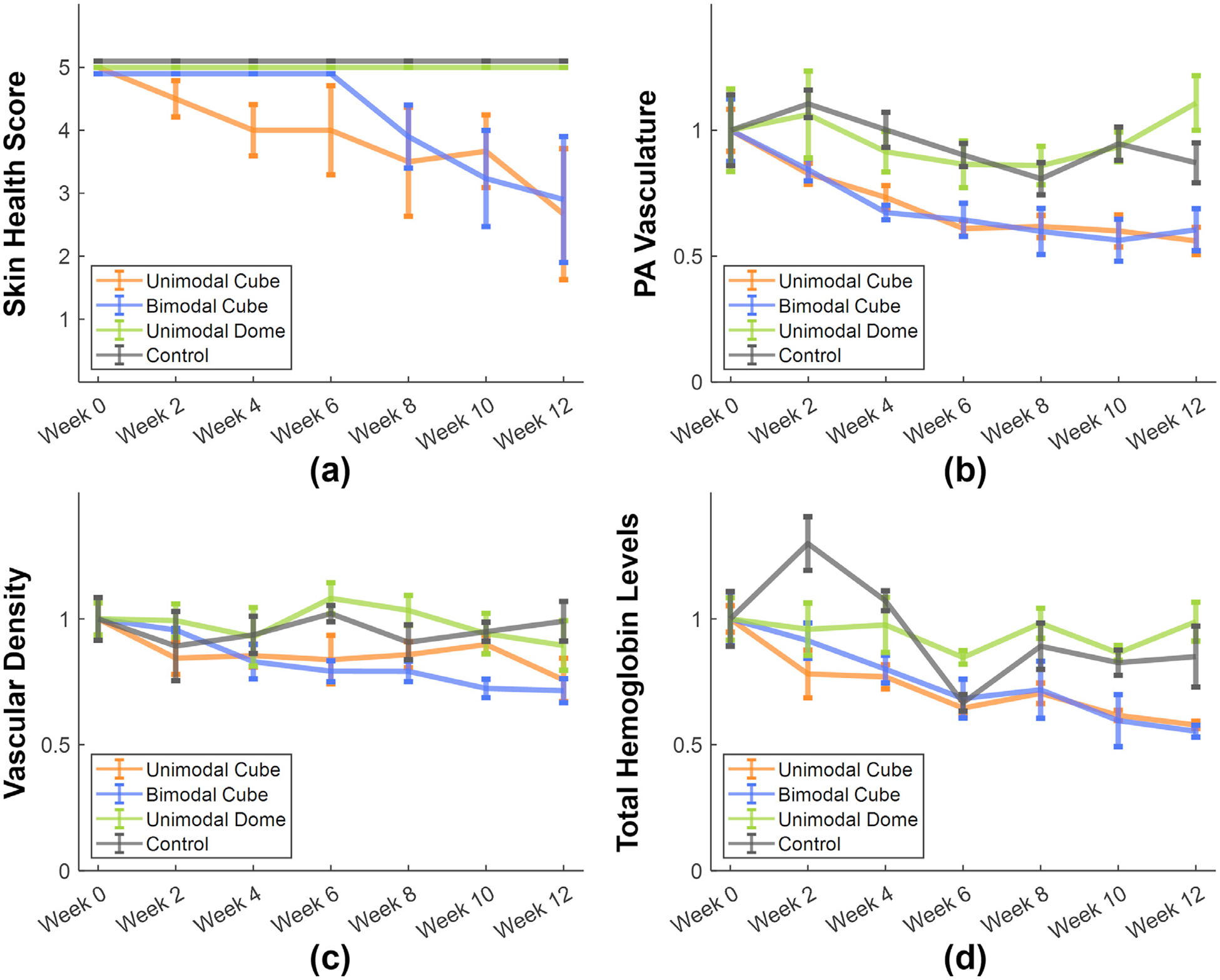
Longitudinal plots of (a) skin health score, (b) photoacoustic vasculature, (c) vascular density and (d) total hemoglobin levels in the skin region, all normalized to the baseline (week 0), from weeks 0 to 12. Orange, blue, olive green and black lines represent the unimodal cube, bimodal cube, unimodal dome and control groups, respectively. Error bars indicate the standard error of the mean.

Examples of segmented 3-D maps of PA vasculature, 2-D maps of enhanced vascular structure and 3-D maps of total hemoglobin for each group at week 0 are presented in Figure 2. PA vasculature showed strong signals throughout the skin region, capturing both capillaries and larger vessels. In contrast, the enhanced vascular structure map provided improved contrast for these major vessels, while the total hemoglobin map primarily highlighted strong signals from major vessels. Longitudinal changes in skin health score, PA vasculature, vascular density and total hemoglobin levels from week 0 to 12, normalized to the baseline (week 0), are illustrated in Figure 3. Throughout the study period, all subjects in both the control (N = 4) and unimodal dome (N = 4) groups maintained healthy skin, with no signs of spots, blisters or dehiscence. In the unimodal cube group (N = 4), three mice exhibited pronounced skin dehiscence and implant exposure by week 12, while one maintained healthy. In the bimodal cube group (N = 4), two mice developed skin dehiscence and implant exposure by week 12, one remained healthy and one mouse died at week 4 before any signs of skin health deterioration were observed. Compared with the unimodal cube group, the bimodal cube group showed a delayed onset of skin health deterioration.

Across all three PA-derived biomarkers, the control and unimodal dome groups exhibited no significant decreases over time. In contrast, the unimodal and bimodal cube groups showed a marked decrease in PA vasculature, vascular density and total hemoglobin levels, suggesting reduced blood volume and vascular integrity post-implantation. These findings are consistent with our hypothesis that elevated mechanical stress results in vascular ischemia and match the observed deterioration in skin health.

### Statistical analysis

**Figure 4.**
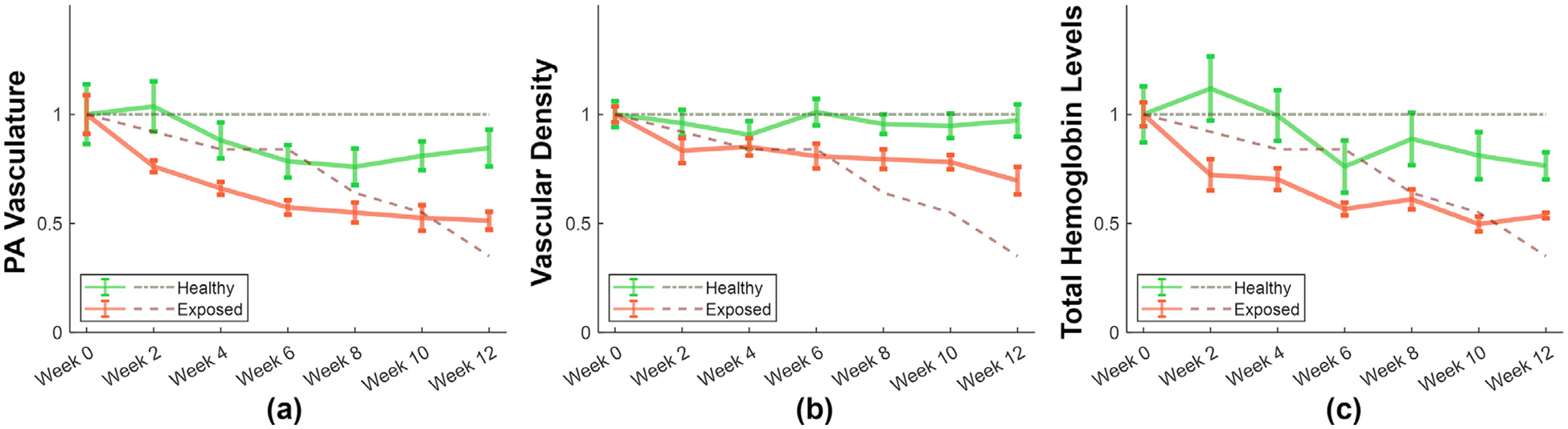
Longitudinal plots of (a) photoacoustic vasculature, (b) vascular density and (c) total hemoglobin levels, all normalized to the baseline (week 0), from weeks 0 to 12. The green and red lines represent the healthy and exposed groups, respectively. Dark green and dark red dashed lines represent the corresponding skin health scores. Error bars indicate the standard error of the mean.

To evaluate the effectiveness of different PA biomarkers, subjects were categorized into two groups: a healthy group (N = 11) and an exposed group (N = 5). Figure 4 presents the longitudinal plot of PA vasculature, vascular density and total hemoglobin levels all normalized to baseline (week 0), from week 0 to week 12, with corresponding skin health scores overlaid as dash lines. In the healthy group, skin health scores remained consistently at 5 throughout the study. In contrast, the exposed group showed a progressive decline, with the average score dropping to 1.75 by week 12. Correspondingly, all three PA biomarkers exhibited a decreasing trend in the exposed group, with reductions of up to 48% by week 12. In the healthy group, no significant decreases were observed, with changes of up to 23% at week 12.

To statistically assess whether the temporal evolution of each PA biomarker differed between groups, a linear mixed effects model with analysis of variance was employed to evaluate the significance of the interaction term. For PA vasculature, the interaction effect was statistically significant (*p* = 0.0013), indicating that the temporal profile differed significantly between healthy and exposed subjects. The interaction for vascular density was also statistically significant (*p* = 0.0129), while the interaction term for total hemoglobin levels approached significance (*p* = 0.0545). These results suggest that PA vasculature is the most sensitive metric for distinguishing exposed from healthy subjects over time and may serve as a more robust early indicator of vascular compromise.

### PA correlation with skin health

As shown in Figure 4, decreases in PA vasculature and total hemoglobin levels in the exposed group preceded the decline in skin health scores. Correlation coefficients and estimated time lags between each PA biomarker and skin health scores for the healthy and exposed groups are summarized in Table 1. In the healthy group, all three PA biomarkers exhibited average correlation coefficients exceeding 0.75, indicating a strong association with skin health. In the exposed group, the correlations were even stronger, with an average correlation coefficient surpassing 0.85. The estimated average time lag for PA vasculature was −3.6 weeks, and −2.8 weeks for total hemoglobin levels, suggesting that reductions in these biomarkers preceded visible skin health deterioration by approximately 2–4 weeks. Vascular density showed an average time lag of +1.2 weeks with a standard deviation of 6.7, indicating no consistent pattern of occurring before or after the onset of skin health deterioration.

To statistically assess the strength of association between each PA biomarker and skin health, single-sample *t*-tests were performed on the cross-correlation coefficients. All three PA biomarkers showed highly significant results, with *p*-values effectively zero, indicating that the correlation coefficients were significantly greater than zero. These results demonstrate that there is a consistent and robust positive relationship between PA-derived biomarkers and skin health across subjects. Among them, PA vasculature and total hemoglobin levels appear to be the most reliable early indicators of impending skin compromise.

### Spatial hemoglobin analysis

**Figure 5.**
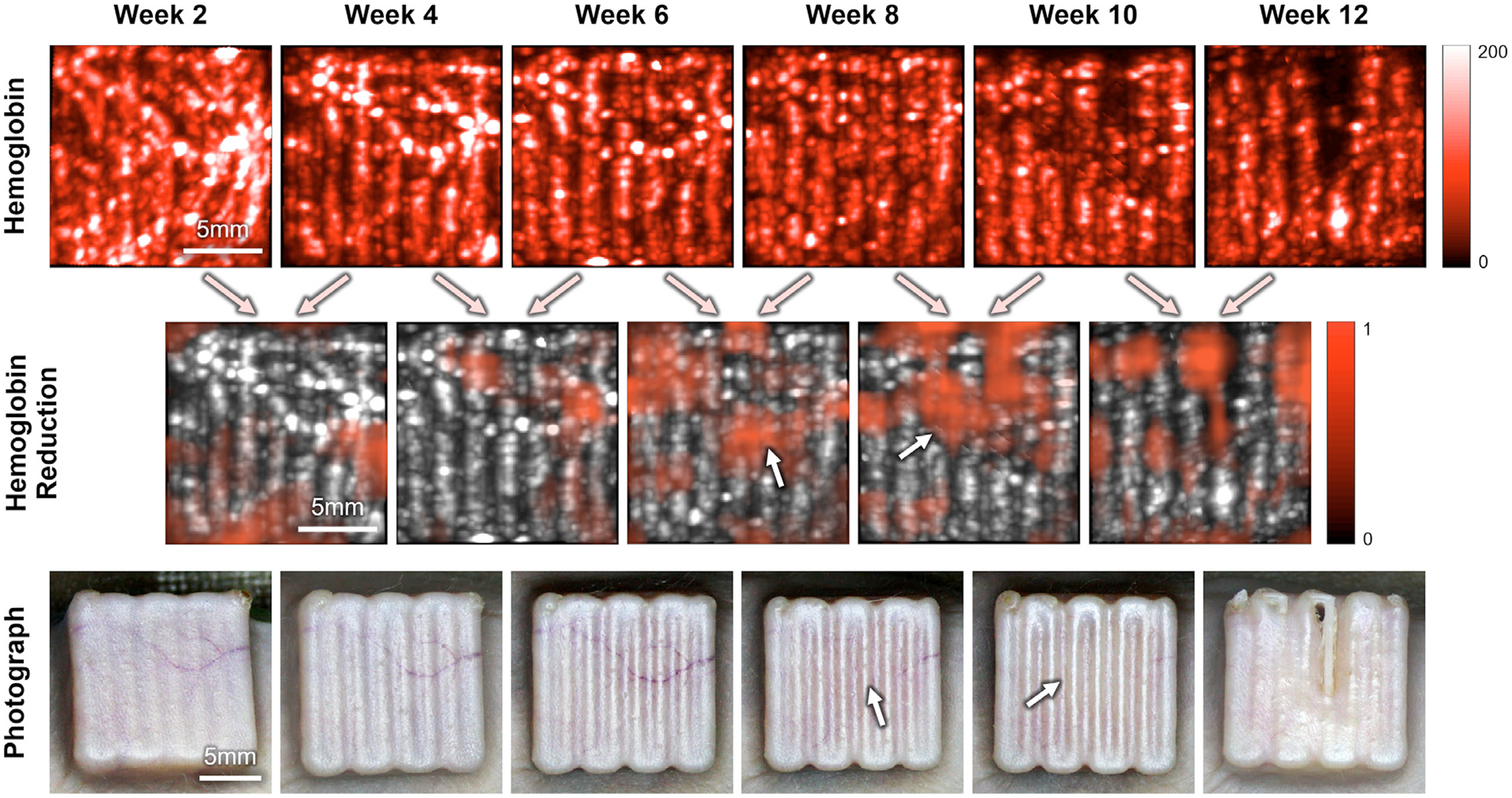
Spatial hemoglobin analysis: (*top row*) 2-D maps of total hemoglobin levels in the skin from weeks 2 to 12, (*middle row*) areas of hemoglobin reduction between consecutive time points (*highlighted in red*) and (*bottom row*) corresponding skin photographs from weeks 2 to 12. White arrows on the hemoglobin reduction maps and photographs indicate regions with visible hemoglobin reduction.

A spatial correlation between regions of decreased total hemoglobin levels and local skin health deterioration was observed in multiple subjects. An illustrative example from the unimodal cube group (Female #1) is shown in Figure 5. The first row presents 2-D maps of total hemoglobin levels in the skin from week 2 to 12. The second row shows regions where total hemoglobin levels decreased between consecutive imaging sessions (marked in red), and the third row displays the corresponding skin photographs. During the experiment, we observed that the skin shifted relative to the implant from week 0 to 2. After week 2, the skin began to integrate with the implant and no further movement was noted. As a result, spatial hemoglobin changes were not analyzed between week 0 and 2 due to this early post-implantation skin movement.

In the total hemoglobin reduction maps, a progressive reduction in total hemoglobin levels was observed at the implant center between weeks 6 and 8, followed by a more pronounced decrease in the upper center region between weeks 8 and 10. These changes correspond with photographic observations, where major blood vessels in the central implant region disappeared between weeks 6 and 8, and the skin in this region became pale between weeks 8 and 10, indicating vascular ischemia. By week 12, the photograph revealed severe skin dehiscence in the upper center region, which spatially aligns with the red-marked region in the hemoglobin reduction map from weeks 8 to 10, where a significant hemoglobin level drop was detected. These findings suggest that US/PA imaging may have the capability to spatially localize at-risk regions before visible dehiscence occurs.

## Discussion

This study demonstrated that US/PA imaging can effectively enable the detection of vascular changes in the skin overlying subcutaneous implants by measuring PA vasculature and total hemoglobin levels. A statistically significant difference in the temporal profile of PA vasculature was observed between healthy and exposed subjects, and all three PA-derived biomarkers showed statistically significant correlations with skin health deterioration. The exposed group exhibited progressive decreases in PA vasculature and total hemoglobin levels, which preceded visible skin necrosis by approximately 2–4 weeks. In contrast, the healthy group maintained stable PA biomarker levels and did not develop skin necrosis. Additionally, a spatial correlation was observed between regions exhibiting reductions in total hemoglobin levels and skin health deterioration. These findings suggest that US/PA imaging can serve as both a temporal and spatial predictor of skin necrosis, enabling early identification of at-risk regions before visible implant exposure occurs. This underscores the potential of PA-derived biomarkers, particularly PA vasculature measured at 532 nm, as non-invasive tools for early detection and monitoring of implant-induced skin necrosis, which could enhance clinical post-implant outcomes and potentially mitigate implant exposure.

Previous studies have qualitatively hypothesized that subcutaneous implants may cause skin necrosis by compromising the vasculature through mechanical stress [22–25]. However, quantitative evidence supporting this hypothesis has been limited. This study establishes a clear, measurable relationship between vascular ischemia and subsequent skin necrosis, reinforcing existing hypotheses while offering novel insights into the temporal and spatial progression of skin necrosis. Furthermore, the findings of this study have direct clinical implications for the design and post-operative monitoring of skin over subcutaneous implants. US/PA imaging could be incorporated into routine clinical practice, enabling the early identification of at-risk regions before irreversible damage occurs. Moreover, refining implant designs based on PA-identified stress-distribution patterns could aid in optimizing implant geometry to minimize vascular compromise and improve long-term tissue integration.

The observed decreases in skin PA vasculature, vascular density and total hemoglobin levels in the exposed group are indicative of vascular ischemia, likely resulting from excessive skin stress imposed by the implant geometry. Finite element modeling [55] suggests that the skin near the four corners and edges of the cube implants experiences significantly higher stress than other regions. This non-uniform stress distribution may contribute to the obstruction and collapse of blood vessels supplying the overlying skin, leading to localized vascular ischemia. The development of skin dehiscence in these groups aligns with biological responses to chronic ischemia, including endothelial dysfunction, impaired wound healing and eventual tissue breakdown. In contrast, the dome implant maintains a more uniform skin interface. Finite element modeling indicates that the dome implant induces only moderately elevated stress at the crest, which remains significantly lower than the stress observed in the cube implants. This reduced stress helps preserve vascular integrity, minimizing the risk of vascular compromise. Consequently, no significant changes in PA measurements or skin health deterioration were observed in the unimodal dome group. These findings highlight the importance of mitigating stress-induced ischemia and emphasize the need for optimized implant designs to reduce vascular compromise.

The bimodal cube implant was designed to enhance tissue and vascular growth through increased porosity [56], and the skin health scores suggest that the bimodal cube group experienced slightly improved skin health outcomes compared with the unimodal cube group. However, no significant differences in PA-derived biomarkers were observed between the two groups. This may be attributed to the limited sample size, especially given that one subject in the unimodal cube group died at week 8 and one subject in the bimodal cube group died at week 4 due to issues unrelated to the implant. Future experiments with larger sample sizes should be conducted to further evaluate the effect of increased porosity on skin health. Additionally, US/PA imaging with greater penetration depth will be employed to track vascular ingrowth and compare the unimodal and bimodal cube groups in more detail.

In Figure 5, the digital photographs show a loss of major vessels in the skin overlying the implant at week 8, followed by signs of vascular ischemia appearing as blanching starting at week 10. These visual observations are consistent with the PA imaging results. While visual monitoring using digital photography can be effective in small animal models with thin skin, its utility is limited in detecting vascular changes in larger animals or human skin, which is typically thicker and more pigmented. In contrast, PA imaging enables quantification of hemoglobin levels in both large and microvascular vessels, allowing for early detection of vascular compromise before surface changes become visible. It also provides volumetric and depth-resolved data, enabling assessment of deeper layers in thicker skin, making it more suitable for future clinical translation.

Multiple vertical ridges are evident in the 2-D maps of hemoglobin levels in Figure 5. These signals do not originate from blood vessels within the skin but rather from the surface of the cube implants. Post-surgery observations revealed that as air within the implant was gradually absorbed, the skin conformed more closely to the implant surface, resulting in a tight and uneven attachment between skin and implant. This made it challenging for the skin segmentation algorithm to perfectly separate the skin region from the implant surface, leading to residual PA signals as visible vertical ridges in the segmentation. This effect was particularly pronounced in female mice, which have thinner skin than males. In future studies, improving the skin segmentation algorithm may help enable more accurate vascular analysis. Additionally, oxygen saturation (sO_2_) was calculated based on the unmixed Hb and HbO_2_ levels from the Vevo LAB. However, no significant changes in sO_2_ were observed across all four groups, nor were there significant differences in sO_2_ changes among the groups. To further investigate this aspect, we plan to conduct a follow-up study focusing specifically on sO_2_ dynamics to better understand potential tissue hypoxia during the progression of skin health deterioration.

While this study provides compelling evidence of US/PA imaging’s potential, it is constrained by the limited sample size and may not be entirely reflective of human skin dynamics. Inter-animal variability in tissue response may influence outcomes, and future studies should aim to validate findings across larger and more diverse populations. Additionally, while US/PA imaging effectively detects changes in blood volume, complementary techniques such as optical coherence tomography or laser Doppler perfusion imaging could provide further validation of ischemic damage [57]. Our group also performed 3-D PA tomography [58–60] and ultrasound shear wave elasticity imaging [61,62] on the experimental mice in this study. Future work will integrate vascular analysis and skin elasticity measurements from those experiments with the findings from this study to provide a more comprehensive analysis, correlating vascular ischemia with the mechanical properties of the skin to better understand the progression of skin health deterioration. Furthermore, future studies should investigate how variations in implant materials, pore structures, mechanical properties, and mitigation strategies influence vascular compromise. Ultimately, longitudinal large animal and human studies will also be necessary to translate these findings into clinical applications.

## Conclusion

This study demonstrated that US/PA imaging can serve as a non-invasive tool for detecting vascular changes and predicting skin health deterioration in response to subcutaneous implants. PA vasculature measured at 532 nm showed a statistically significant difference between the subjects with healthy skin and those with implant exposure. All three PA-derived vascular biomarkers exhibited strong correlations with skin health, with decreases in signal preceding visible skin necrosis by 2–4 weeks. Additionally, spatial hemoglobin analysis suggested a potential association between localized reductions in total hemoglobin levels and regions that later developed skin necrosis. These findings highlight the critical role of implant geometry on vascular integrity, with cube implants inducing vascular ischemia due to localized stress, while the dome implant preserved the overall skin vasculature. Despite the study’s limited sample size, the results suggest that US/PA imaging could aid in guiding implant design and enable early detection of implant-induced skin necrosis. Future work should focus on validating these findings in larger studies and exploring clinical applications for post-operative monitoring and intervention.

## Figures and Tables

**Table 1:** Correlation coefficients and time lags between photoacoustic vasculature, vascular density, total hemoglobin levels and skin health scores, along with the corresponding *p*-values from single-sample *t*-tests. The ± values represent standard deviations.

	Healthy group		Exposed group		
	Correlation coefficient	*p*-value	Correlation coefficient	*p*-value	Time lag (weeks)
PA vasculature	0.77 ± 0.08	2.44 × 10^−11^	0.85 ± 0.12	8.75 × 10^−5^	−3.6 ± 2.6
Vascular density	0.81 ± 0.08	8.48 × 10^−12^	0.87 ± 0.09	2.92 × 10^−5^	1.2 ± 6.7
Hemoglobin level	0.82 ± 0.07	4.20 × 10^−12^	0.85 ± 0.11	5.45 × 10^−5^	−2.8 ± 2.3

## Data Availability

The data that support the findings of this study are available from the corresponding author upon reasonable request.
